# Global prevalence and molecular characteristics of three clades within hepatitis B virus subgenotype C2: Predominance of the C2(3) clade in South Korea

**DOI:** 10.3389/fmicb.2023.1137084

**Published:** 2023-03-09

**Authors:** Dong Hyun Kim, Yu-Min Choi, Junghwa Jang, Bum-Joon Kim

**Affiliations:** ^1^Department of Microbiology and Immunology, College of Medicine, Seoul National University, Seoul, Republic of Korea; ^2^Department of Biomedical Sciences, College of Medicine, Seoul National University, Seoul, Republic of Korea; ^3^Liver Research Institute, College of Medicine, Seoul National University, Seoul, Republic of Korea; ^4^Cancer Research Institute, College of Medicine, Seoul National University, Seoul, Republic of Korea; ^5^Seoul National University Medical Research Center (SNUMRC), Seoul, Republic of Korea

**Keywords:** hepatitis B virus (HBV), subgenotype C2, C2(3), South Korea, sV184A

## Abstract

Hepatitis B Virus (HBV) genotypes reflect geographic, ethical or clinical traits and are currently divided into 10 genotypes (A–J). Of these, genotype C is mainly distributed in Asia, is the largest group and comprises more than seven subgenotypes (C1–C7). Subgenotype C2 is divided into three phylogenetically distinct clades, C2(1), C2(2), and C2(3), and is responsible for most genotype C infections in three East Asian nations, including China, Japan, and South Korea, which are major HBV endemic areas. However, despite the significance of subgenotype C2 with regard to clinical or epidemiologic aspects, its global distribution and molecular characteristics remain largely unknown. Here, we analyze the global prevalence and molecular characteristics between 3 clades within subgenotype C2 using 1,315 full genome sequences of HBV genotype C retrieved from public databases. Our data show that almost all HBV strains from South Korean patients infected with genotype C belong to clade C2(3) within subgenotype C2 [96.3%] but that HBV strains from Chinese or Japanese patients belong to diverse subgenotypes or clades within genotype C, suggesting clonal expansion of a specific HBV type, C2(3), among the Korean population. Our genome sequence analysis indicated a total of 21 signature sequences specific to the respective clades C2(1), C2(2), and C2(3). Of note, two types of four nonsynonymous C2(3) signature sequences, sV184A in HBsAg and xT36P in the X region, were detected in 78.9 and 82.9% of HBV C2(3) strains, respectively. In particular, HBV strains C2(3) versus C2(1) and C2(2) show a higher frequency of reverse transcriptase mutations related to nucleot(s)ide analog (NA) resistance, including rtM204I and rtL180M, suggesting an increased possibility of C2(3) infection in those with NA treatment failure. In conclusion, our data show that HBV subgenotype C2(3) is extremely prevalent in Korean patients with chronic HBV infection, which is distinct from two other East Asian nations, China and Japan, where diverse subgenotypes or clades within genotype C coexist. This epidemiologic trait might affect distinct virological and clinical traits in chronic HBV patients in Korea, where exclusively C2(3) infection is predominant.

## Introduction

The discovery of hepatitis B virus (HBV) led to the first vaccine not prepared through tissue culture but that was initially directly prepared from plasma, with a later recombinant vaccine produced from yeast (da Fonseca, 2010). Despite an effective vaccine, HBV infection remains a severe global health issue, with more than 240 million people being chronic carriers and approximately 786,000 patient deaths annually worldwide due to HBV-related diseases, including liver cirrhosis (LC) and hepatocellular carcinoma (HCC; Schweitzer et al., 2015; Guvenir and Arikan, 2020). HBV is an endemic disease in South Korea (Korean National Health and Nutrition Survey of 2011); the prevalence of hepatitis B virus surface antigen (HBsAg) positivity is 3.4% in males and 2.6% in females (Yim and Kim, 2019).

HBV, which belongs to the *Hepadnaviridae* family, is an enveloped virus with a partially double-stranded DNA genome of approximately 3.2 kb in length that includes 4 overlapping open reading frames (ORFs) encoding the surface protein (S), core protein (C), polymerase (Pol), and HBx protein (X; Liang, 2009; Datta et al., 2012). HBV has been characterized into 10 genotypes, A to J (Sunbul, 2014), which are subdivided into more than 30 subgenotypes (Kurbanov et al., 2010; Shi et al., 2012). Each subgenotype has been reported to show distinct geographical patterns and clinical traits (Liu et al., 2021). Genotype C is the oldest and most common extant genotype and is endemic in the Asia-Pacific region (Velkov et al., 2018; Kyaw et al., 2020). Compared to genotype B, genotype C exhibits higher HBV replication capacity and tends to result in chronic infection, which may lead to the development of LC and/or HCC (Chan et al., 2003; Gao et al., 2007). In addition, incomplete response to IFN therapy and higher levels of mutations have been reported in genotype C HBV infections (Kao et al., 2000).

Genotype C is further divided into many subgenotypes, C1 to C11 (McNaughton et al., 2020). Subgenotype C2 is endemic in Far Eastern countries, including Korea, China and Japan, which are close geographically and similar in historical and social-economic aspects (Lin et al., 2019). Rather diverse genotypes are observed in China and Japan in addition to subgenotype C2, whereas an extremely high prevalence of subgenotype C2 has been reported in Korea (Kim et al., 2007; Cho et al., 2009). More recent studies have shown that subgenotype C2 can be further divided into three phylogenetically distinct clades, C2(1), C2(2), and C2(3) (Yin et al., 2019; McNaughton et al., 2020), but the correlation of these clades with geographic and clinical traits has not yet been analyzed.

Studies on the correlation between regional-specific genotypes and epidemiologic traits may provide an explanation for specific clinical or virological issues in local population (Lin et al., 2019). In Korea, there are higher rates of naturally occurring HBV variants related to clinical severity compared to other countries, and occult infection or potential antiviral drug resistance and a higher frequency of IFN therapy failure occur even with intensive medical care (Yim and Kim, 2019). In this study, we investigated the prevalence of three clades within subgenotype C2, i.e., C2(1), C2 (2), and C2 (3), in East Asian countries. A total of 683 whole-genome sequences of HBV subgenotype C2 of 1,315 HBV genotype C retrieved from public databases were used to analyze overall mutation rates and signature mutations specific for each clade.

## Materials and methods

### Acquisition of HBV genome sequence data

**Figure 1:**
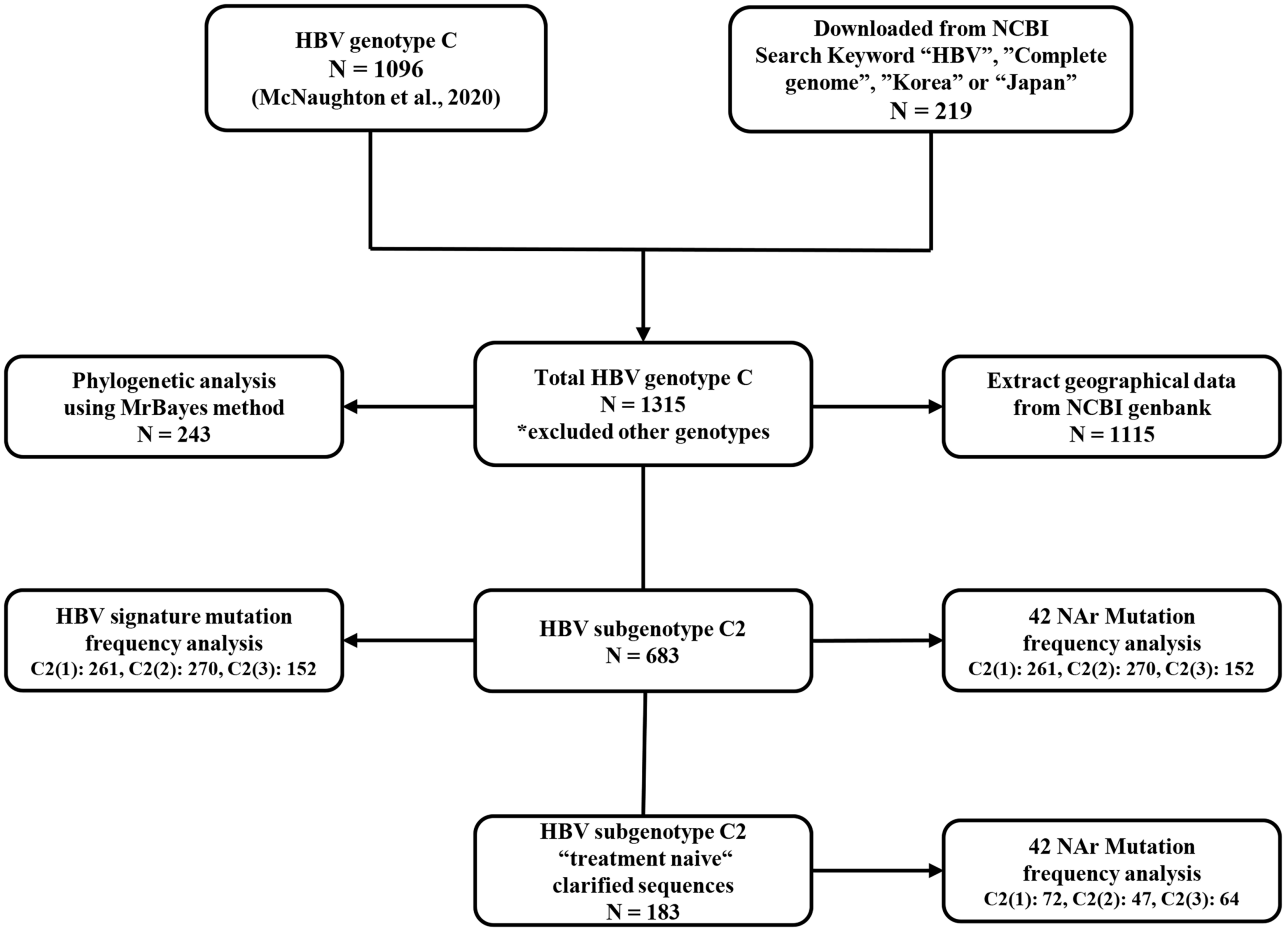
Flowchart of dataset preparation and data composition for each analysis.

A total of 1,315 HBV genome sequences of genotype C were used in this study. With 1,096 sequences from a previous research dataset (McNaughton et al., 2020), additional Korean and Japan sequences from NCBI^1^ databases with the keywords “HBV,” “complete genome,” “Korea,” or “Japan” were downloaded, and the final 219 sequences were genotyped using phylogenetic analysis. HBV genotype C and genotype A sequence (accession no. AB116076, AB116080, AB452979) alignments were fitted to 3,215 and 3,221 bp, respectively. The genotyped datasets used in this manuscript were constructed in FASTA format (Supplementary Table S1). A flowchart of the database configuration in each analysis is shown (Figure 1).

### Phylogenetic analysis and genotyping

Alignments were constructed using MAFFT with the FFT-NS-2 algorithm in Geneious Prime software (2022.1.1, Biomatters, Inc., New Zealand), and all indels in alignments were deleted. A total of 1,315 sequences were genotyped by phylogenetic analysis using the approximate maximum likelihood method with the FastTree program in Geneious Prime software (2022.1.1, Biomatters, Inc., New Zealand; Supplementary Figure S1). Then, 243 genotype C sequences, including 40, 34, and 36 well-branched sequences of each C2(1), C2(2), and C2(3) clade, were extracted. A phylogenetic tree with the 243 sequences was constructed by the MrBayes program (Huelsenbeck and Ronquist, 2001) using the MCMC method and GTR substitution model. The topology of the phylogenetic tree was confirmed by the maximum likelihood method with the Tamura-Nei model (Tamura and Nei, 1993) using the MEGA X program (Tamura et al., 2021). An accession number list organized by subgenotype, including geographical information, was constructed in Excel format (Supplementary Table S2).

### Genetic distance calculation

A total of 683 subgenotype C2 sequences, 261, 270, and 152 sequences belonging to subgenotypes C2(1), C2(2), and C2(3), respectively, were used for genetic distance calculations. Consensus sequences were extracted using the majority rule from each subgenotype and aligned using Geneious Prime software (2022.1.1, Biomatters, Inc., New Zealand). Pairwise distance between sequences and the consensus in each subgenotype was calculated using the MEGA X program (Tamura et al., 2021).

### Mutation and signature sequence analysis

The number of preC/Core and BCP mutations and polymorphism rates for the rt269 region were analyzed for each clade (Tong et al., 2007; Jeong et al., 2021). The amino acid composition of each clade was analyzed for 42 potential analog-resistant (NAr)-related amino acid mutations identified in a previous study (Liu et al., 2010). The total mutation rate for all 42 NAr sites was calculated by dividing the number of sequences in each clade multiplied by 42 by the number of mutations in each clade. By examining the source of the obtained accession number, we checked whether the patients with respective sequences experienced antiviral treatment before serum extraction. Then, the sequences were further classified into four categories: “treatment naïve,” “antiviral treatment,” “not mentioned,” and “unpublished” (Supplementary Table S3). The frequency of 42 potential NAr-related amino acid mutations with sequences from 183 “treatment-naïve” patients was further analyzed. A polymorphism that uniquely appeared with a frequency of ≥70% in each subgenotype was defined as a signature sequence. Signature sequences were found throughout the HBV genome, and the nucleotide composition ratio (A, T, G, C) with other clades was compared for each signature sequence. Due to the characteristics of the overlapping genome of HBV, nonsynonymous or synonymous mutations of two proteins can occur simultaneously within one signature sequence, and both were considered. Gaps in alignment or strains that were not properly sequenced were excluded.

### Analysis of two signature mutations specific to C2(3), sV184A in HBsAg and xT36P in the X region in Korean patients

**Figure 2:**
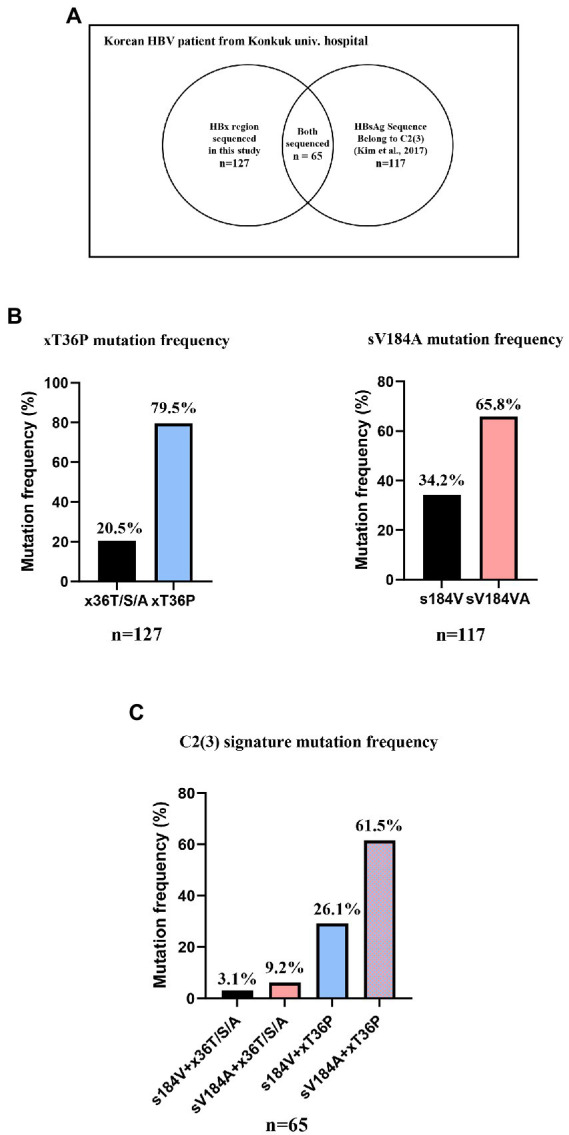
The frequency of two signature mutations specific for C2(3), sV184A and xT36P, from Korean chronic HBV infection patients from Konkuk university hospital. **(A)** A Venn diagram of 131 previously studied RT sequences and 127 HBx sequences generated in this study. **(B)** A total of 79.5% of Korean HBV C2(3) patients carried the xT36P mutation in HBx, and 65.8% of the patients carried sV184A in HBsAg. **(C)** A total of 65 DNA sequences of the HBsAg and HBx regions were accessible, and 61.5% showed both C2(3) signature sequences.

Serum DNA samples from 127 Korean HBV patients who visited Konkuk University Hospital were extracted as previously described (Kim et al., 2017). For the frequency of xT36P in HBx, the HBx region of 127 DNA samples was newly sequenced using the following primers (FW: CTC TGC CGA TCC ATA CTG CGG AA, RV: TTA ACC TAA TCT CCT CCC CCA; Sugauchi et al., 2001). The frequency of sV184A in HBsAg was analyzed using the overlapping HBsAg region of reverse transcriptase sequences from the 131 Korean patients analyzed in our previous study (accession nos. KX264864~KX264922, KX264792~KX264863; Kim et al., 2017). As shown in Figure 2A, 65 patient DNA samples overlapped between the 131 samples with the reverse transcriptase sequenced in a previous study and the HBx sequences from 127 samples in this study. The frequency of the sV184A and xT36P sequences from the overlapping 65 patients was calculated. The 127 HBx sequences are shown in Supplementary Table S4. As previously extracted virion DNA from isolates was used in this study, informed consent and waiver of informed consent were not required by the IRB of the hospital (IRB1012-131-346).

### Phylogenetic analysis based on reverse transcriptase sequences from 131 Korean patients

A 1,032 bp fragment of HBV DNA reverse transcriptase from 131 Korean patients was aligned to reverse transcriptase sequences extracted from the 683 subgenotype C2 sequence dataset using the MAFFT method, as described above. A phylogenetic tree was generated using the maximum likelihood method with the Tamura-Nei model (Tamura and Nei, 1993).

### Statistical analysis

Statistical analysis was conducted using GraphPad Prism 8.0 software for Windows, GraphPad Software, San Diego, California United States, www.graphpad.com. Statistical significance of the number of amino acid differences was calculated by ANOVA (analysis of variance). For analysis of mutation sites, wild-type and mutated sequences were compared using the chi-square test, and the significance calculated is shown in related figures and tables.

## Results

### Global prevalence of subgenotypes C2(1), C2(2), and C2(3) using HBV genome-based phylogenetic analysis in three east Asian countries

**Figure 3:**
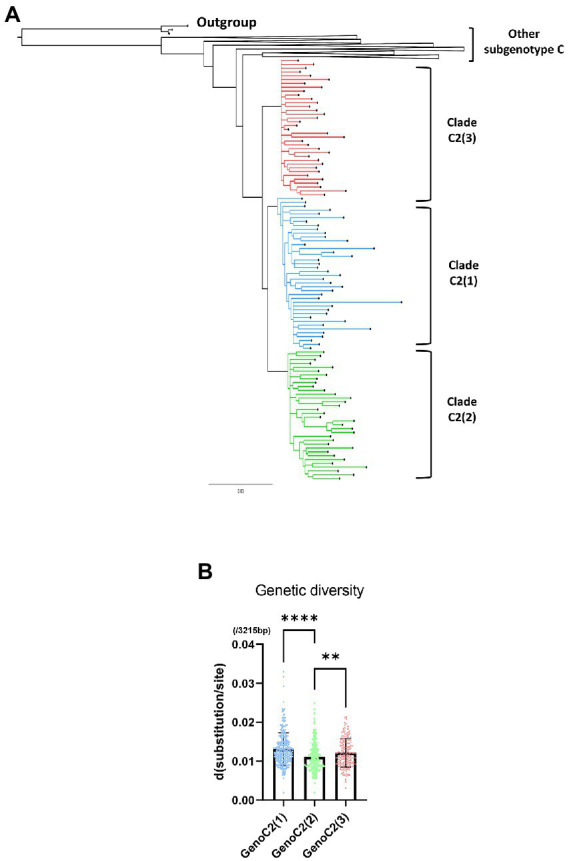
A phylogenetic tree of hepatitis B virus genotype C based on 243 genome sequences constructed using MrBayes. For phylogenetic analysis between three clades within subgenotype C2, 40 of 260 sequences from C2(1), 34 of 271 from C2(2) and 36 of 152 from C2(3) were selected. **(A)** Three clades of subgenotype C2 are shown in blue [C2(1)], green [C2(2)], and red [C2(3)]. **(B)** Comparison of HBV genetic diversity among clades C2(1), C2(2), and C2(3). *p < 0.05; **p < 0.01; ***p < 0.001; ****p < 0.0001.

A phylogenetic tree was created using a total of 1,315 HBV genotype C sequences, including 1,096 from a previous research dataset (McNaughton et al., 2020) and an additional 219 from “Korean” or “Japanese” patients downloaded for use in this study through the approximate maximum likelihood method (Supplementary Figure S1). The phylogenetic tree showed that 893 of the 1,315 sequences belonged to subgenotype C2 and were separated into three distinct clades, C2(1) (299 sequences), C2(2) (321 sequences), and C2(3) (273 sequences). We isolated 40 C2(1), 36 C2(2), and 34 C2(3) sequences in each clade and 133 sequences in other subgenotype C sequences from the tree. To clarify the branching between clades, a phylogenetic tree was created with 243 selected genotype C sequences, including 110 subgenotype C2 [40 C2(1), 36 C2(2), and 34 C2(3)] sequences, using the MrBayes method (Figure 3A). Our phylogenetic analysis showed separation of three phylogenetically distinctive subclades within subgenotype C2, consistent with previous findings (McNaughton et al., 2020). This separation and topology was also demonstrated in a tree produced using the maximum-likelihood method (Supplementary Figure S2). C2(1) and C2(3) form two independent clusters with C2(2) branching from C2(1), suggesting recent evolution of C2(2) from C2(1).

**Figure 4:**
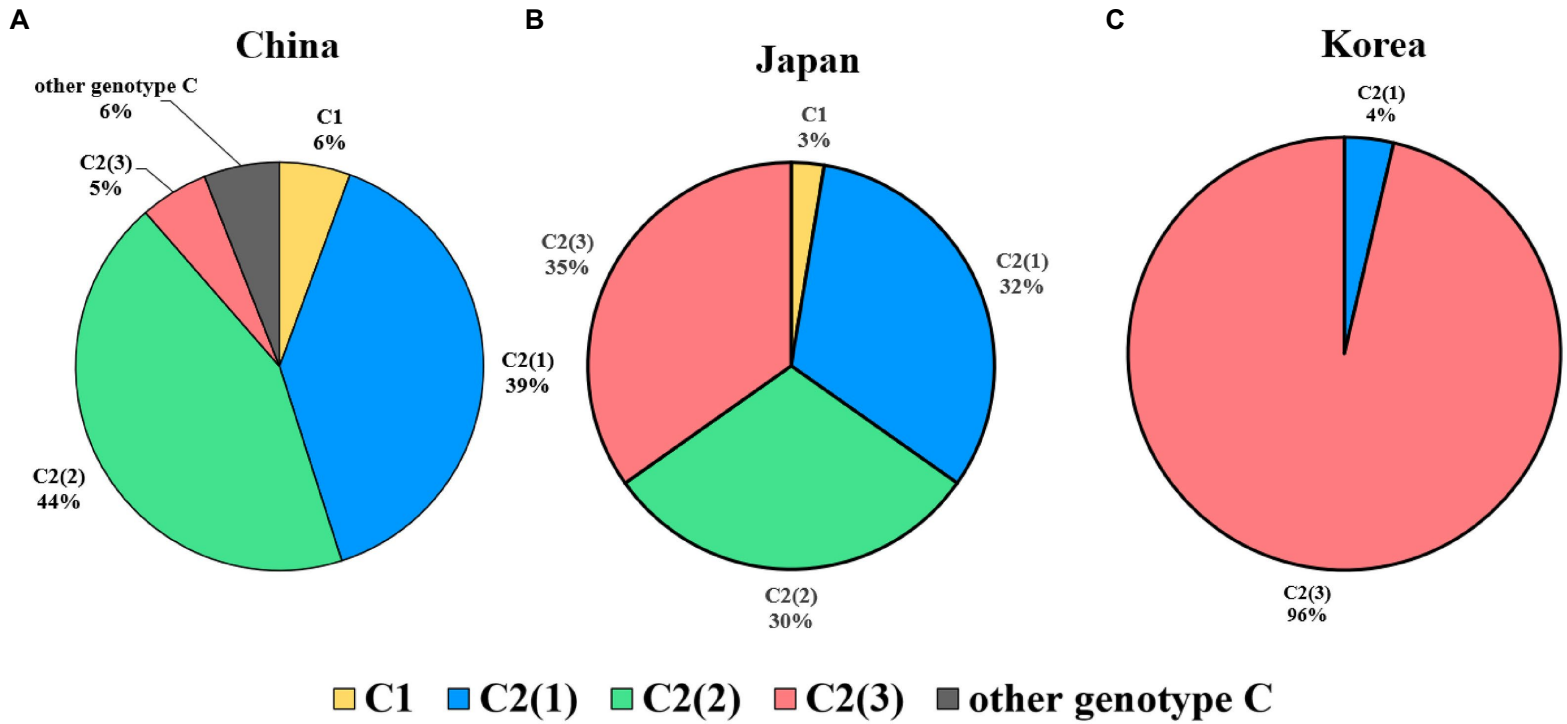
Global prevalence of three clades within hepatitis B virus subgenotype C2. **(A)** In China, C2(2) is the most prevalent genotype, followed by C2(1). **(B)** In Japan, the three subgenotypes are distributed at approximately 30%. **(C)** In Korea, the subgenotype C2(3) is overwhelmingly dominant and shows lower genotypic diversity than in the other two countries.

Regarding genetic diversity, the C2(2) HBV sequence exhibited the lowest average substitution per site (0.013 ± 0.004), which was statistically significant when compared to C2(1) (0.011 ± 0.004; *p* < 0.0001), and C2(3) (0.012 ± 0.004; *p* value < 0.01; Figure 3B). Geographical distribution analysis of 1,115 sequences available with country information among the genotype C sequence pool revealed subgenotype C2 strains to be predominant in three countries of East Asia: China (457/517 strains, 88.4%), Japan (111/114 strains, 97.4%) and South Korea (108/108 strains, 100%; Supplementary Table S5). Overall, geographical distribution at the clade level within subgenotype C2 indicated that subgenotype C2(3) is extremely dominant in South Korea (104/108, 96.3%) but that its prevalence is relatively low in China (28/457, 5.4%) and Japan (40/114, 35.1%; Figure 4). Together, our data indicate that subgenotype C2 infections are predominant in three nations of East Asia, China, Japan and South Korea, and that C2(3) infection is responsible for most chronic hepatitis B patients in South Korea.

### Comparison of preC/C mutation (G1896A) and basal core promoter double mutations (A1762T and G1764A) among C2(1), C2(2), and C2(3)

**Figure 5:**
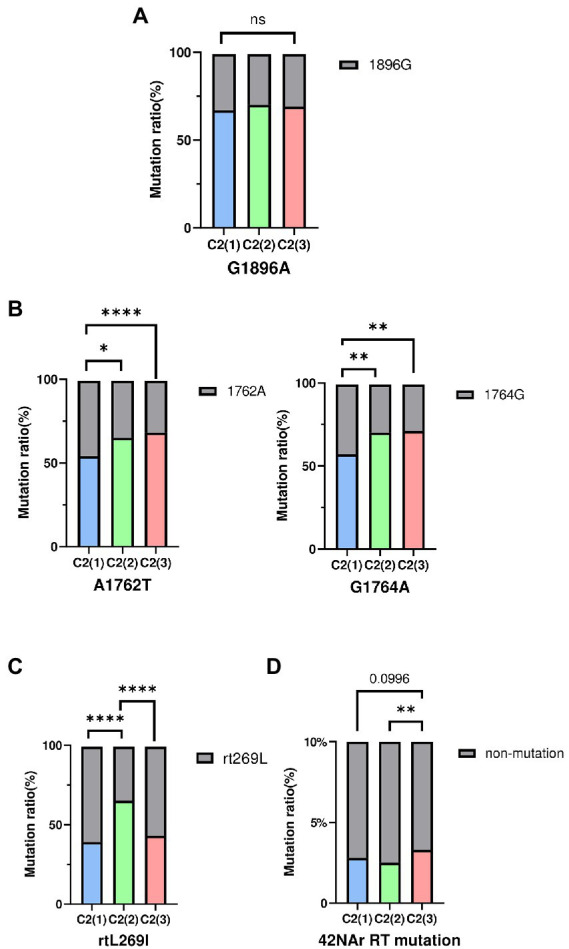
The frequency of HBV mutations between three clades. **(A)** Comparisons of preC/C mutation (G1896A) **(B)** Comparisons of BCP mutation (A1762T, G1764A). **(C)** Comparisons of reverse transcriptase polymorphism rtL269I. **(D)** Comparisons of total 42NAr mutation sites. Categorical variables were tested using the Chi-squared test. ns, nonsignificant, ^*^p < 0.05; ^**^p < 0.01; ^***^p < 0.001; ^****^p < 0.0001.

HBV strains belonging to subgenotype C2 have been reported to have higher frequencies of preC mutation (G1896A) and basal core promoter (BCP) double mutations (A1762T and G1764A), which are associated with abolition of HBeAg and its reduced expression, respectively (Park and Lee, 2016). Thus, mutation frequency was evaluated between clades C2(1), C2(2), and C2(3) using a total of 683 subgenotype C2 strains selected from the HBV sequence pool: C2(1) (*n* = 261), C2(2) (*n* = 270), and C2(3) (*n* = 152). Our data showed there was no significant difference between strains of the 3 clades with respect to frequency of the preC/C mutation (G1896A; Figure 5). However, for BCP double mutations A1762T and G1764A, strains belonging to C2(2) or C2(3) exhibited significantly higher frequency compared with C2(1), despite no significant difference between C2(2) and C2(3), consistent with the previous finding of higher frequency of BCP mutation in chronic HBV infection patients in South Korea (Lee et al., 2015). The frequency of the rtL269I polymorphism associated with the development of liver disease was significantly higher in clade C2(2) than in C2(1) and C2(3).

### HBV strains belonging to subgenotype C2(3) vs. C2(1) and C2(2) are more prone to some reverse transcriptase mutations related to nucleos(t)ide analog resistance

As genotype C HBV strains have been reported to have a higher frequency of reverse transcriptase (RT) mutations related to nucleos(t)ide analog (NAr) resistance (Kim et al., 2017), the amino acid mutation frequency of 42 potential NAr positions in HBV RT sequences (Liu et al., 2010) was evaluated between clades C2(1), C2(2) and C2(3) in a total of 683 subgenotype C2 strains. C2(3) showed a higher frequency of mutations at a total of 42 potential NAr positions than C2(1) or C2(2) (*p* < 0.05; Figure 5D). In particular, the frequency of YMDD-motif mutation at rt204 corresponding to the primary drug resistance mutation (category 1) was detected at a significantly higher rate in C2(3) than in C2(1) or C2(2) (*p* < 0.001; Table 1). The frequency of the L180M mutation, corresponding to a secondary drug resistance mutation, and the H126C/Y/Q mutation, a putative ADV NAr, were observed at a significantly higher frequency in C2(3) compared to C2(1) or C2(2) (*p* < 0.05, *p* < 0.001, respectively; Nakajima et al., 2021). The T128N/I mutation frequency among the putative NAr mutations was higher in C2(1) than in C2(2) or C2(3) (*p* < 0.01), and in the pretreatment mutation column, the mutation frequency of Y124H and D134E/N/C was higher in C2(1) than in other clades (*p* < 0.0.05, *p* < 0.001, respectively). To evaluate NAr mutation frequency in treatment-naïve patients of subgenotype C2, potential NAr mutation frequency was further analyzed using sequences of the 183 selected “treatment-naïve” patients, 72 C2(1), 47 C2(2), and 64 C2(3) sequences (Supplementary Table S6). Our data showed that of the putative NAr mutations, F221Y and N238D/S/H were significantly higher in C2(3) than in other clades (*p* < 0.01, *p* < 0.05, respectively). Among the pretreatment mutations, D134E/N/C was most common in C2(1) (*p* < 0.01), and C2(3) was statistically significant in N139K/H (*p* < 0.05). As shown in 683 subgenotype C2 strains, C2(3) also showed a significantly higher frequency of mutations at a total of 42 potential NAr positions compared to C2(1) or C2(2) in 183 selected “treatment-naïve” patients (*p* < 0.0001).

**Table 1:** Occurrence of NAr mutation in reverse transcriptase of hepatitis B virus genotype C2 by clade.

Reverse transcriptase	Drug resistance	C2(1) (*n* = 261)	C2(2) (*n* = 270)	C2(3) (*n* = 152)	*P*-value
Primary drug resistance					
I169 L/T	ETV	0% (0/261)	1.11% (3/267)	0.66% (1/151)	
A181T/V	LMV, ADV	0.77% (2/259)	1.48% (4/265)	0.66% (1/151)	
T184A/C/F	ETV	1.15% (3/258)	0.37% (1/269)	1.32% (2/150)	
A194T	ADV	0% (0/261)	0% (0/270)	0.66% (1/151)	
S202C/G/I	ETV	0.38% (1/260)	0.37% (1/269)	0.66% (1/151)	
M204I/V	LMV/ETV	5.75% (15/246)	7.43% (25/245)	11.84% (27/125)	0.0003
N236T	ETV	0.38% (1/260)	0.37% (2/268)	0% (0/152)	
M250I/L/V	ETV	1.15% (3/258)	0.74% (2/268)	0.66% (1/151)	
Secondary mutation					
L80I	LMV	3.1% (8/252)	3% (12/259)	3.9% (6/146)	
L180M	LMV/ETV	4.6% (12/249)	4.4% (12/258)	10.5% (16/136)	0.0209
Putative NAr mutation					
S53N	LMV	0.8% (2/259)	1.1% (3/267)	0.7% (1/151)	
T54N	ADV	0.4% (1/260)	0.4% (1/269)	0% (0/152)	
L82M/V	LMV	0% (0/261)	(0/270)	0.7% (1/151)	
V84M/I	ADV	0.8% (2/259)	0.4% (1/269)	0 (0/152)	
S85A	ADV	(2/259)	(1/269)	(2/150)	
I91L	LMV	0.4% (1/260)	0.7% (2/268)	0.7% (1/151)	
H126C/Y/Q	ADV	2.7% (7/254)	1.1% (3/267)	8.6% (13/139)	0.0002
T128N/I	LMV	10.3% (27/234)	3.7% (10/260)	6.6% (9/143)	0.0085
N139D	LMV	6.1% (16/245)	10.4% (28/242)	7.2% (11/141)	
R/W153Q	LMV	1.1% (3/258)	1.1% (3/267)	0%(0/152)	
F166L	LMV	0% (0/261)	0.4% (1/269)	0% (0/152)	
V191I/D	LMV, ADV	0.4% (1/260)	0% (0/270)	0% (0/152)	
A200V	LMV	0.8% (2/259)	1.1% (3/267)	2.0% (3/149)	
V207I	LMV	3.1% (8/253)	1.1% (2/268)	3.3% (5/147)	
S213T	ADV	1.9% (5/256)	2.2% (6/264)	3.9% (6/146)	
V214A	ADV	0.4% (1/260)	0.4% (1/269)	0.7% (1/151)	
Q215P/S/H	LMV, ADV	0.8% (2/259)	1.1% (3/267)	0% (0/152)	
L217R	ADV	0.8% (2/259)	0% (0/270)	1.3% (2/150)	
E218D	ADV	0% (0/261)	0.7% (2/268)	0.7% (1/151)	
F221Y	ADV	6.5% (17/244)	8.9% (24/247)	9.2% (14/138)	
L229G/V/W	LMV	4.2% (11/250)	4.1% (11/259)	3.9% (6/146)	
I233V	ADV	0% (0/261)	0.4% (1/269)	0.7% (1/151)	
P237H	ADV	0% (0/261)	0.4% (1/269)	0% (0/152)	
N238D/S/H	ADV	5.7% (15/246)	5.9% (16/254)	9.2% (14/138)	
Y245H	ADV	0% (0/261)	0.4 (1/269)	0% (0/152)	
S/C256G	LMV, ETV	0% (0/261)	0% (0/270)	0% (0/152)	
Pretreatment mutation					
T38A		2.3% (6/255)	1.9% (5/265)	2.0% (3/149)	
Y124H		15.2% (40/221)	7.4% (20/250)	10.5% (16/136)	0.0144
D134E/N/C		19.2% (50/211)	7.8% (21/249)	10.5% (15/137)	0.0002
N139K/H		6.1% (16/245)	10.7% (29/241)	7.2% (11/141)	
I224V		6.9% (18/243)	7.8% (21/249)	7.2% (11/141)	
R242A		0.4% (1/260)	0.4% (1/269)	0% (0/152)	
Ratio of total 42 NAr mutation		2.8% (299/10663)	2.5% (281/11059)	3.3% (201/6183)	0.0312

* Counted except for gap, ambiguous site.

### Identification of signature mutations specific to the three clades C2(1), C2(2), and C2(3)

We identified signature mutations with significantly higher frequencies that are nonsynonymous and synonymous and specific to each clade. A total of 21 types of signature nonsynonymous (8) and synonymous mutations (13) specific for the respective clades were found (Supplementary Table S7). The signature mutations were present in the preS (4 types), S (1 types), X (3 types), PreC/C (2 types) and Pol (16 types) regions. Of these, 1 (1 NS mutation), 13 (3 NS mutations), and 7 (4 NS mutations) mutation types were specific to C2(1), C2(2), and C2(3), respectively (Table 2). L116V mutation in HBx was the only detected signature mutation of C2(1) (81.6% of strains). In C2(2), we observed 3 nonsynonymous mutations of 13 signature mutations: S314P in the Pol region (93.7%), F321L in the Pol spacer region (91.1%), and A90V in the preS1 region (89.3%). Of the 7 signature mutations in C2(3), 4 are signature nonsynonymous mutations: sV184A mutation in HBsAg (78.9%), T/S/A36P in HBx (82.9%), H81N mutation in the Pol terminal protein region (97.4%) and T272A in the Pol spacer region (76.3%).

**Table 2:** Nonsynonymous mutation sites and their incidence rate in each clade of subgenotype C2.

C2 subclade	Site			Clade	A	C	G	T	Total
Genomic number	Region	Nucleotide	Amino acid						
C2(1) signature sequences									
1719	HBx	T1719G	L116V	C2(1)	0	0	218 (81.6%)	48 (18.4%)	261
				C2(2), C2(3)	0	0	15 (2.4%)	412 (97.6%)	422
C2(2) signature sequence									
**31**	preS2	T399C		C2(2)	0	252 (93.7%)	0	16 (5.9%)	268
	Pol (Spacer)	T940C	S314P	C2(1), C2(3)	0	14 (3.4%)	0	399 (96.6%)	413
**52**	preS2	T420C		C2(2)	0	245 (91.1%)	0	22 (8.2%)	267
	Pol (Spacer)	T961C	F321L	C2(1), C2(3)	0	19 (4.6%)	0	394 (95.4%)	413
**3,116**	preS1	A269T	A90V	C2(2)	1 (0.4%)	28 (10.4%)	0	241 (89.3%)	270
	Pol (Spacer)	C810T		C2(1), C2(3)	1 (0.2%)	403 (97.6%)	0	9 (2.2%)	413
C2(3) signature sequence									
**705**	Reverse transcriptase	T1614C(rtT588C)		C2(3)	0	120 (78.9%)	0	32 (21.1%)	152
	HBsAg	T551C	sV184A	C2(1), C2(2)	0	10 (1.9%)	0	520 (97.9%)	530
**1,479**	RNase H	G/A2388C		C2(3)	16 (10.5%)	126 (82.9%)	6 (3.6%)	4 (2.6%)	152
	HBx	A/T/G106C	T/S/A36P	C2(1), C2(2)	334 (62.9%)	23 (4.3%)	104 (19.6%)	68 (12.8%)	529
**2,547**	Terminal protein	C241A	H81N	C2(3)	148 (97.4%)	3 (2.0%)	0	1 (0.7%)	152
				C2(1), C2(2)	28 (5.3%)	501 (94.4%)	0	2 (0.4%)	531
**3,120**	Spacer	A814G	T272A	C2(3)	20 (13.2%)	1 (0.7%)	116 (76.3%)	14 (9.2%)	151
	preS1	A273G		C2(1), C2(2)	509 (95.9%)	7 (1.3%)	11 (2.1%)	3 (0.6%)	530

* Counted except for gap, ambiguous site.

### Differentiation at the clade level of Korean patients with chronic HBV infection *via* sequence analysis of the HBV reverse transcriptase region

**Figure 6:**
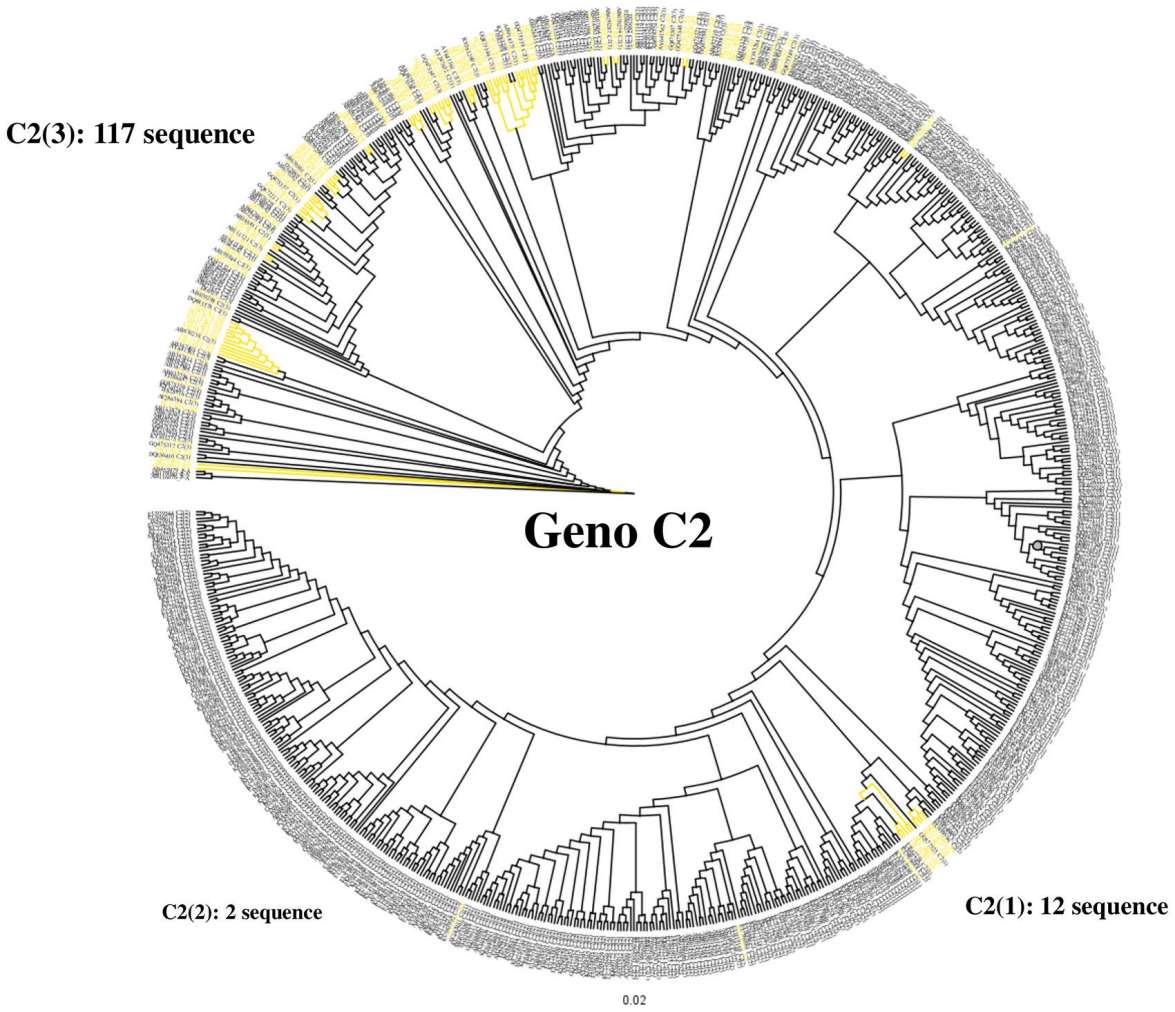
Differentiation at the clade level of 131 Korean chronic patients *via* phylogenetic analysis of the HBV reverse transcriptase region. Phylogenetic tree of reverse transcriptase from HBV subgenotype C2. Distribution of 131 Korean patients by alignment with 683 previously constructed subgenotype C2 reverse transcriptase sequences. Most Korean patients (89.3%) are infected with subgenotype C2(3). Korean patient sequences are shown in yellow branches.

To confirm the dominance of C2(3) in Korean patients with chronic HBV infection, we evaluated the clade distribution using 131 HBV RT sequences previously reported by our laboratory (Kim et al., 2017; Figure 6). Phylogenetic analysis using the maximum likelihood method showed that among the 131 patients, all belong to C2(3) (117/131 patients, 89.3%), except for 12 patients with C2(1) and 2 with C2(2), further supporting our finding of the dominance of C2(3) in South Korea. Of the 117 Korean patients infected with C2(3), 77 (65.8%) carried the sV184A mutation in the overlapping HBsAg region and 101 (79.5%) the xT36P mutation in HBx (Figure 2). Of a total of 65 patients for whom sequence information for both HBsAg and HBx regions was available, the proportion of those carrying both sV184A and xT36P was highest at 61.5% (40/65), followed by those with xT36P alone, at 26.1% (17/65), sV184A alone, at 9.2%, and s184V/+x36T/S/A, at 3.1%.

## Discussion

HBV subgenotype C2 is responsible for most genotype C infections in three nations of East Asia, namely, China, Japan and South Korea, which are major HBV endemic areas (Cho et al., 2009; Wang et al., 2010; Demarchi et al., 2022). Subgenotype C2 is reported to have unique virological and clinical traits distinct from other genotypes and subgenotypes, including higher virulence, higher BCP or preC/C mutation frequency, and lower response to IFN-I therapy (Lin and Kao, 2011; Ito et al., 2018; Tang et al., 2018). Phylogenetic analysis based on entire HBV genome sequences to date indicates the existence of three distinct clades, C2(1), C2(2), and C2(3), within subgenotype C2 (McNaughton et al., 2020). However, studies regarding the global prevalence and molecular characteristics of these three distinct clades remain largely unknown. In this study, we for the first time determined the global prevalence and molecular characteristics of three distinct clades, C2(1), C2(2) and C2(3), within subgenotype C2.

First, we found differences between China, Japan and South Korea with regard to the clade distribution of subgenotype C2. C2(3) was found to be exclusively predominant in South Korea, distinct from China and Japan, showing coexistence of the three clades despite a discrepancy in their distribution (Figure 4). This epidemiologic finding may provide a likely explanation for the distinct findings of several studies using Korean cohorts, including a higher frequency of NAr or BCP mutation (Kim, 2014), the presence of rarely encountered HBV mutation types (Kim et al., 2017), and a higher prevalence of HBeAg-negative HCC patients (Jang et al., 2022).

Second, we identified 21 types of signature sequences specific for C2(1), C2(2) and C2(3), which may be used for differentiation of the 3 clades as genetic markers (Table 2). In particular, 8 types of nonsynonymous signature sequences might influence the distinct virological and clinical traits of each clade. Future studies should focus on nonsynonymous signature sequences to elucidate the underlying mechanism associated with the distinct virological and clinical traits of each clade.

Third, we found that compared to the other two clades, i.e., C2(1) and C2(2), C2(3) showed a higher frequency of NAr mutations, even in rt204 and rt180, which are related to primary and secondary drug resistance, respectively (Table 1). This suggests that C2(3) infection may lead to enhanced NA treatment failure compared to the other two clades. Indeed, our previous study using a cohort of treatment-naïve patients with chronic HBV infection revealed a higher prevalence of naturally occurring NAr mutations in Korea compared to other areas, including China (Kim et al., 2017). Our previous LNA-based RT–PCR assay also showed a higher prevalence of rt204I region YMDD variants, particularly in Korean HCC patients (Choe et al., 2019). Higher rates of relapse in Korean chronic HBV infection patients with HBeAg seroconversion after lamivudine treatment have also been found, supporting the above hypothesis (Song et al., 2000).

Fourth, despite no significant difference in the frequency of preC mutation, the BCP double mutation frequency was significantly higher in chronic HBV patients infected with C2(3) or C2(2) than in those infected with C2(1) (Figure 5). Given the relationships of BCP double mutation with liver disease progression and HBeAg-negative infection (Kim, 2014), the possibility that C2(3) or C2(2) vs. C2(1) may cause more advanced liver diseases, including HCC in HBeAg-negative chronic HBV patients, cannot be excluded (Zhang et al., 2010; Alexopoulou and Karayiannis, 2014). In fact, a recent study using a Korean cohort reported that HCC is more prevalent in HBeAg-negative patients without liver cirrhosis than in HBeAg-positive patients without liver cirrhosis, which is distinct from other studies showing that HBeAg is not an independent risk factor for HCC (Jang et al., 2022). This issue should be further assessed in future studies using Korean cohorts.

In conclusion, our data show that HBV subgenotype C2(3) is extremely prevalent in Korean patients with chronic HBV infection, which is distinct from two other East Asian countries, China and Japan, where diverse subgenotypes or clades within genotype C coexist. In addition, we found that HBV strain C2(3) shows a higher frequency of RT mutations related to NAr, including rtM204I and rtL180 M, than strains C2(1) and C2(2), suggesting an increased possibility of C2(3) infection in those with NA treatment failure. This epidemiologic trait might affect distinct virological and clinical traits in patients with chronic HBV infection in Korea, where C2(3) infection is exclusively predominant.

## Data availability statement

The datasets presented in this study can be found in online repositories. The names of the repository/repositories and accession number(s) can be found in the article/Supplementary material.

## Ethics statement

The studies involving human participants were reviewed and approved by the Institutional Review Board of Seoul National University Hospital (1012-131-346). Written informed consent for participation was not required for this study in accordance with the national legislation and the institutional requirements.

## Author contributions

DK and B-JK designed the study. B-JK interpreted the research and wrote the first draft of the manuscript. DK performed the data analysis and revised the manuscript. Y-MC and JJ supported the data analysis and revised the manuscript. All authors contributed to the article and approved the submitted version.

## Funding

This research was supported by the National Research Foundation of Korea (NRF) and funded by the Ministry of Education (grant no. NRF-2022R1A2B5B01001421). This research was also supported by a grant from the Korea Health Technology R&D Project through the Korea Health Industry Development Institute (KHIDI), as funded by the Ministry of Health and Welfare, Republic of Korea (grant no. HI22C0476). Y-MC, DK, and JJ received a scholarship from the BK21-plus education program provided by the National Research Foundation of Korea.

## Conflict of interest

The authors declare that the research was conducted in the absence of any commercial or financial relationships that could be construed as a potential conflict of interest.

## Publisher’s note

All claims expressed in this article are solely those of the authors and do not necessarily represent those of their affiliated organizations, or those of the publisher, the editors and the reviewers. Any product that may be evaluated in this article, or claim that may be made by its manufacturer, is not guaranteed or endorsed by the publisher.

## Footnotes

^1^ https://www.ncbi.nlm.nih.gov
