# Supplementary material for: Global prevalence and molecular characteristics of three clades within hepatitis B virus subgenotype C2: Predominance of the C2(3) clade in South Korea
Source: Front Microbiol. 2023 Mar 9;14:1137084. doi: 10.3389/fmicb.2023.1137084 (PMC10033913; doi:10.3389/fmicb.2023.1137084)
Supplement: Supplementary file 3 [file Data_Sheet_3.docx]

**Supplementary Figure S1**. A phylogenetic tree constructed using 1315 sequences by the approximate maximum likelihood method in the FastTree program. Three clades of subgenotype C2 are shown in blue (C2(1)), green (C2(2)) and red (C2(3)).

**
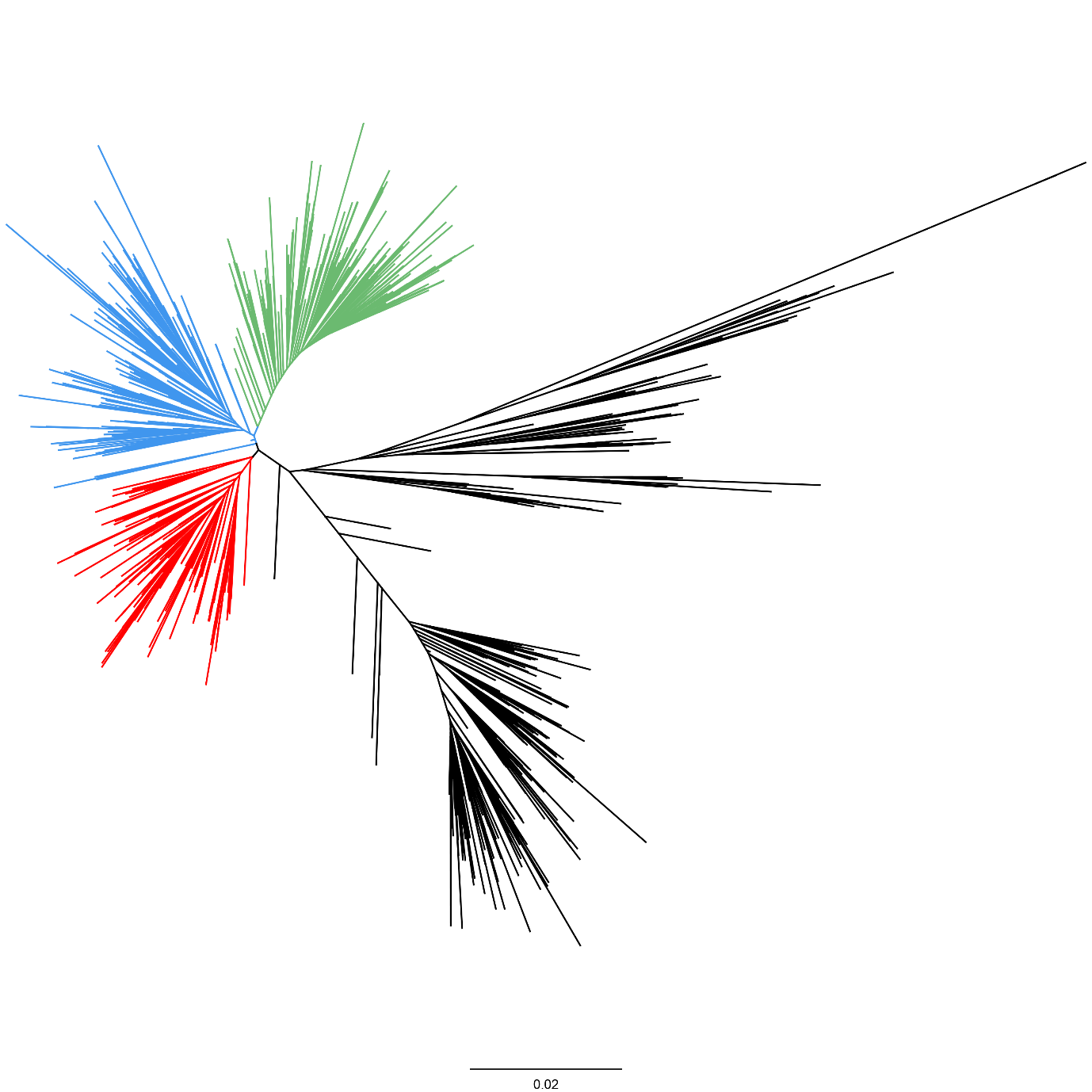
**


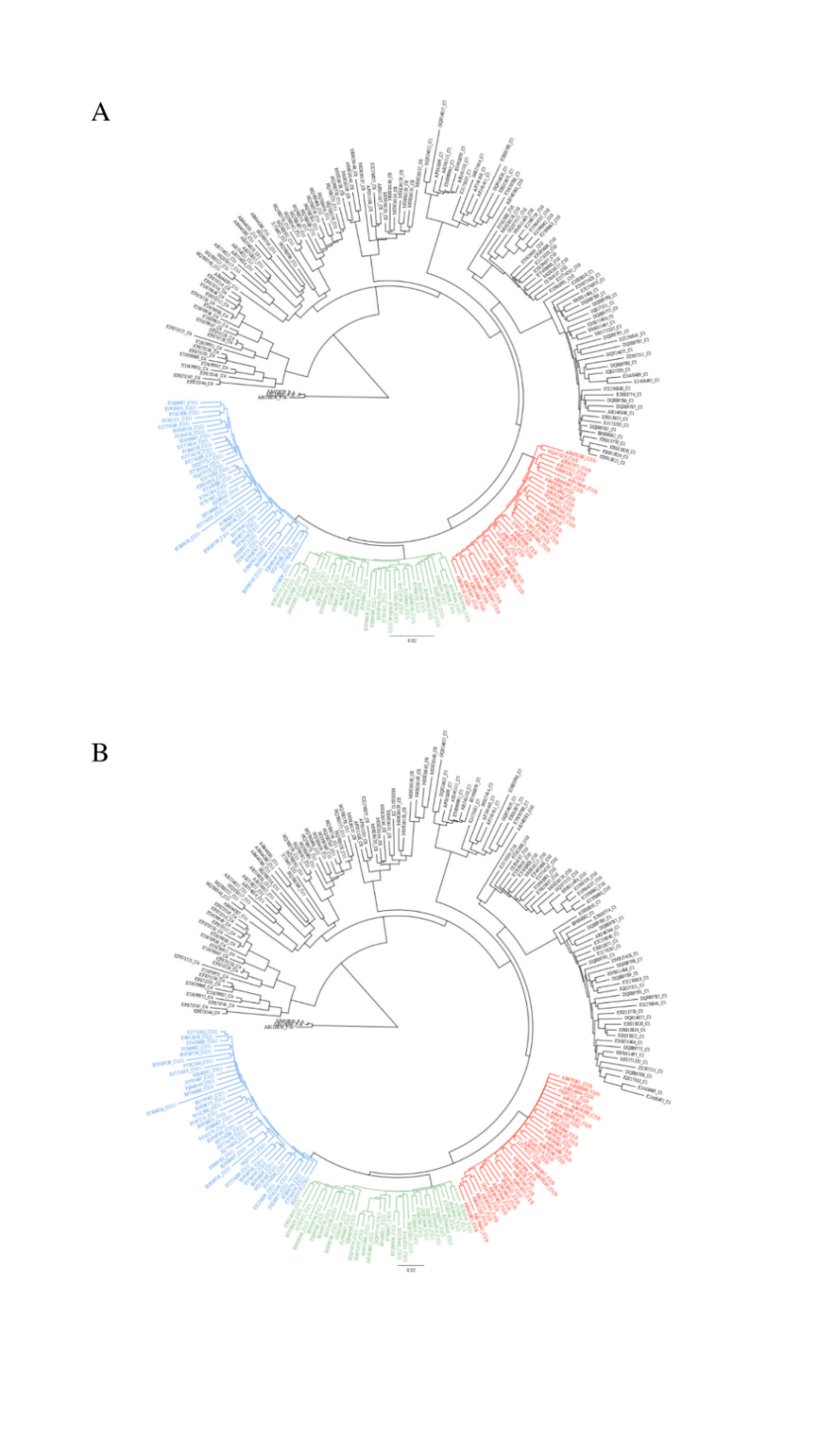
 **Supplementary Figure S2**. Comparison of phylogenetic trees between the MrBayes method and the maximum likelihood method. Three clades of subgenotype C2 are shown in blue (C2(1)), green (C2(2)) and red (C2(3)). **(A)** A phylogenetic tree constructed using the MrBayes method. **(B)** A phylogenetic tree constructed using the maximum likelihood method

**Supplementary Table S1.** A total of 1315 HBV genotype C sequences aligned in FASTA format.

**Supplementary Table S2.** A list of genotypes and geographical information of 1315 accession numbers in Excel format.

**Supplementary Table S3.** A list of accession numbers classified according to therapy status by referring to each source paper in Excel format. Each sequence was classified into one of four categories: “treatment naïve”, “antiviral treatment”, “not mentioned”, and “unpublished”.

**Supplementary Table S4**. HBx alignment sequenced in this study from 127 patients in FASTA format.

**Supplementary Table S5.** Regional specificity of HBV genotype C based on 1115 genome sequences. Subgenotype C2 is the most prevalent in East Asian countries. Over 96% of HBV genotype C sequences recorded in Korea belong to subgenotype C2(3).

| **Genotype** | **C1** | **C2(1)** | **C2(2)** | **C2(3)** | **C4** | **C5** | **C8** | **C10** | **C11** | **Total** |
| --- | --- | --- | --- | --- | --- | --- | --- | --- | --- | --- |
| China | 5.6% (29) | 39.8% (206) | 43.1% (223) | 5.4% (28) |  | 0.6% (3) | 2.3% (12) | 3.1% (16) |  | 517 |
| Japan | 2.6% (3) | 32.5% (37) | 29.8% (34) | 35.1% (40) |  |  |  |  |  | 114 |
| Korea |  | 3.7% (4) |  | 96.3% (104) |  |  |  |  |  | 108 |
| etc. | 68.4% (257) | 6.4% (24) | 1.6% (6) | 3.5% (13) | 5.6% (21) | 4.0% (15) | 1.3% (5) | 1.3% (5) | 8% (30) | 376 |
| Total | 289 | 271 | 262 | 186 | 21 | 18 | 17 | 21 | 30 | 1115 |

| **Reverse**  **transcriptase** | **Drug resistance** | | **C2(1) (n=72)** | **C2(2) (n=47)** | **C2(3) (n=64)** | **p value** |
| --- | --- | --- | --- | --- | --- | --- |
| **Primary Drug resistance** | | |  |  |  |  |
| I169 L/T | | ETV | 0% (0/72) | 0% (0/47) | 0% (0/64) |  |
| A181T/V | | LMV, ADV | 0% (0/72) | 0% (0/47) | 0% (0/64) |  |
| T184A/C/F | | ETV | 0% (0/72) | 0% (0/47) | 0% (0/64) |  |
| A194T | | ADV | 0% (0/72) | 0% (0/47) | 0% (0/64) |  |
| S202C/G/I | | ETV | 0% (0/72) | 2.1% (1/47) | 0% (0/63) |  |
| M204I/V | | LMV/ETV | 2.8% (2/70) | 0% (0/47) | 6.3% (4/64) | 0.1798 |
| N236T | | ETV | 0% (0/72)0 | 0% (0/47) | 0% (0/64) |  |
| M250I/L/V | | ETV | 0% (0/72) | 0% (0/47) | 0% (0/64) |  |
| **Secondary mutation** | | |  |  |  |  |
| L80I | | LMV | 0% (0/72) | 0% (0/47) | 1.6% (1/64) |  |
| L180M | | LMV/ETV | 0% (0/72) | 0% (0/47) | 1.6% (1/64) |  |
| **Putative NAr mutation** | | |  |  |  |  |
| S53N | | LMV | 0% (0/72) | 0% (0/47) | 0% (0/64) |  |
| T54N | | ADV | 0% (0/71) | 0% (0/47) | 0% (0/64) |  |
| L82M/V | | LMV | 0% (0/72) | 0% (0/47) | 1.6% (1/64) |  |
| V84M/I | | ADV | 0% (0/72) | 0% (0/47) | 0% (0/64) |  |
| S85A | | ADV | 0% (0/72) | 0% (0/47) | 0% (0/64) |  |
| I91L | | LMV | 0% (0/72) | 0% (0/47) | 0% (0/64) |  |
| H126C/Y/Q | | ADV | 2.8% (2/72). | 2.1% (1/47) | 9.4% (6/64) | 0.1221 |
| T128N/I | | LMV | 0% (0/70) | 0% (0/45) | 0% (0/60) |  |
| N139D | | LMV | 1.5% (1/67) | 0% (0/47) | 0% (0/58) |  |
| R/W153Q | | LMV | 2.8% (2/72) | 0% (0/47) | 0% (0/64) |  |
| F166L | | LMV | 0% (0/70) | 2.1% (1/47) | 0% (0/64) |  |
| V191I/D | | LMV, ADV | 0% (0/70) | 0% (0/47) | 0% (0/64) |  |
| A200V | | LMV | 1.4% (1/72) | 2.1% (1/47) | 0% (0/64) |  |
| V207I | | LMV | 0% (0/69) | 0% (0/45) | 0% (0/62) |  |
| S213T | | ADV | 1.4% (1/71) | 0% (0/47) | 1.6% (1/63) |  |
| V214A | | ADV | 0% (0/70) | 0% (0/47) | 0% (0/64) |  |
| Q215P/S/H | | LMV, ADV | 0% (0/70) | 0% (0/47) | 0% (0/64) |  |
| L217R | | ADV | 0% (0/70) | 0% (0/47) | 0% (0/64) |  |
| E218D | | ADV | 0% (0/70) | 0% (0/47) | 0% (0/64) |  |
| F221Y | | ADV | 4.2% (3/71) | 0% (0/47) | 15.6% (10/64) | 0.0032 |
| L229G/V/W | | LMV | 4.2% (3/72) | 0% (0/47) | 0% (1/62) |  |
| I233V | | ADV | 0% (0/70) | 0% (0/47) | 0% (0/64) |  |
| P237H | | ADV | 0% (0/70) | 0% (0/47) | 0% (0/64) |  |
| N238D/S/H | | ADV | 1.4% (1/72) | 2.1% (1/47) | 10.9% (7/64) | 0.0217 |
| Y245H | | ADV | 0% (0/70) | 0% (0/47) | 0% (0/64) |  |
| S/C256G | | LMV, ETV | 0% (0/70) | 0% (0/47) | 0% (0/64) |  |
| **Pretreatment mutation** | | |  |  |  |  |
| T38A | |  | 1.4% (1/69) | 2.2% (1/46) | 3.1% (2/64) |  |
| Y124H | |  | 6.9% (5/72) | 0% (0/47) | 10.9% (7/64) | 0.0700 |
| D134E/N/C | |  | 24.4% (12/69) | 0% (0/46) | 7.8% (5/64) | 0.0066 |
| N139K/H | |  | 1.5% (1/67) | 0% (0/47) | 9.4% (6/64) | 0.0183 |
| I224V | |  | 8.3% (6/72) | 2.1% (1/47) | 7.0% (4/63) |  |
| R242A | |  | 0% (0/71) | 0% (0/47) | 0% (0/64) |  |
| Ratio of total  42 NAr mutation | |  | 1.4%  (41/3024) | 0.5%  (9/1974) | 2.0%  (54/2688) | <0.0001 |

**Supplementary Table S6.** Frequency of NAr mutations in sequences of treatment-naïve patients

*Counted except for gap, ambiguous site

**Supplementary Table S7.** Synonymous mutation site and their incidence rate in each clade of subgenotype C2

| **C2 subclade** | **Site** | | | **Clade** | | **A** | **C** | | **G** | **T** | **Total** |  |
| --- | --- | --- | --- | --- | --- | --- | --- | --- | --- | --- | --- | --- |
| **Genomic number** | **Region** | **Nucleotide** | |  |  |  |  |  |  |  |  |  |
| **C2(2) signature sequence** | | | | | | | | | | | |  |
| **915** | Pol  (RT) | | G1824A | C2(2) | | 0 | 252  (93.7%) | 0 | | 16  (5.9%) | 268 |  |
|  |  |  |  | C2(1), C2(3) | | 0 | 14  (3.4%) | 0 | | 399  (96.6%) | 413 |  |
| **993** | Pol  (RT) | | A1902G | C2(2) | | 0 | 245  (91.1%) | 0 | | 22  (8.2%) | 267 |  |
|  |  |  |  | C2(1), C2(3) | | 0 | 19  (4.6%) | 0 | | 394  (95.4%) | 413 |  |
| **1053** | Pol  (RT) | | G1962A | C2(2) | | 1  (0.4%) | 28  (10.4%) | 0 | | 241  (89.3%) | 270 |  |
|  |  |  |  | C2(1), C2(3) | | 1  (0.2%) | 403  (97.6%) | 0 | | 9  (2.2%) | 413 |  |
| **1218** | Pol  (Rnase H) | | C2127T | C2(2) | | 5  (1.9%) | 5  (1.9%) | 0 | | 259  (95.9%) | 269 |  |
|  |  |  |  | C2(1), C2(3) | | 11  (2.7%) | 384  (93.0%) | 1  (0.2%) | | 16  (3.9%) | 413 |  |
| **1221** | Pol  (Rnase H) | | A2130T | C2(2) | | 15  (5.4%) | 6  (2.2%) | 4  (1.4%) | | 252  (90.6%) | 278 |  |
|  |  |  |  | C2(1), C2(3) | | 355  (86.0%) | 9  (2.2%) | 7  (1.7% | | 41  (9.9%) | 412 |  |
| **1230** | Pol  (Rnase H) | | G2139C | C2(2) | | 5  (1.9%) | 236  (87.4%) | 28  (10.4%) | | 0 | 269 |  |
|  |  |  |  | C2(1), C2(3) | | 1  (0.2%) | 4  (1.0%) | 407  (98.5%) | | 0 | 412 |  |
| **1727** | HBx | | A354G | C2(2) | | 18  (6.7%) | 0 | 252  (93.3%) | | 0 | 270 |  |
|  |  |  |  | C2(1), C2(3) | | 342  (82.8%) | 2  (0.5%) | 67  (16.2%) | | 0 | 411 |  |
| **2201** | Core | | T388C | C2(2) | | 1  (0.8%) | 245  (90.7%) | 0 | | 23  (8.5%) | 269 |  |
|  |  |  |  | C2(1), C2(3) | | 0 | 23  (5.6%) | 0 | | 390  (94.4%) | 413 |  |
| **2290** | Core | | C477T | C2(2) | | 3  (1.1%) | 13  (4.8%) | 4  (1.5%) | | 249  (92.2%) | 269 |  |
|  |  |  |  | C2(1), C2(3) | | 16  (3.9%) | 358  (86.7%) | 2  (0.5%) | | 36  (8.7%) | 412 |  |
| **2699** | Pol  (Terminal Protein) | | G/T393A | C2(2) | | 191  (70.7%) | 61  (22.6%) | 11  (4.1%) | | 6  (2.2%) | 269 |  |
|  |  |  |  | C2(1), C2(3) | | 19  (4.6%) | 11  (2.7%) | 223  (54.0%) | | 160  (38.7%) | 413 |  |
| **C2(3) signature sequence** | | | | | | | | | | | |  |
| **912** | Reverse  Transcriptase | | G1821A  (rtG795A) | C2(3) | | 147  (96.7%) | 0 | 4  (2.6%) | | 1  (0.7%) | 152 | |
|  |  |  |  | C2(1), C2(2) | | 76  (14.3%) | 0 | 450  (84.7%) | | 5  (0.9%) | 531 | |
| **2684** | Terminal  protein | | C378T | C2(3) | | 0 | 2  (1.3%) | 1  (0.7%) | | 149  (98.0%) | 152 | |
|  |  |  |  | C2(1), C2(2) | | 1  (0.2%) | 484  (91.1%) | 0 | | 46  (8.7%) | 531 | |
| **2699** | Terminal  Protein | | A/C/G393T | C2(3) | | 0 | 3  (2.0%) | 0 | | 149  (98.0%) | 152 | |
|  |  |  |  | C2(1), C2(2) | | 210  (39.5%) | 69  (13.0%) | 234  (44.1%) | | 17  (3.2%) | 530 | |

*Counted except for gap, ambiguous site
